# Transpalpebral electrical stimulation for the treatment of retinitis pigmentosa: study protocol for a series of N-of-1 single-blind, randomized controlled trial

**DOI:** 10.1186/s13063-024-07933-0

**Published:** 2024-01-27

**Authors:** Wei Zhou, Ziyang Huang, Kai Xu, Yamin Li, Xiaoyu Li, Jiaxian Li, Yu Jin, Torkel Snellingen, Lina Liang

**Affiliations:** 1 Department of Eye Function Laboratory, Eye Hospital, China Academy of Chinese Medical Sciences, Beijing, China; 2 Beijing Research Institute of Vision Science & Sekwa Eye Hospital, Beijing, China

**Keywords:** Transpalpebral electrical stimulation, Microcurrent stimulation, Retinitis pigmentosa, N-of-1 trial

## Abstract

**Background:**

Retinitis pigmentosa (RP) is an inherited disease characterized by a progressive loss of rod photoreceptors of the eye, leading to irreversible blindness. To date, to our knowledge, no clinical prospective studies have been undertaken that could document the effect of interventions that could reverse or reduce the progression of this disease. The application of microcurrent stimulation (ES) of the eye in the treatment of chronic eye diseases such as glaucoma and age-related macular degeneration has been used over several decades and has been reported to have beneficial effects to reduce the progression of these blinding diseases and has been supported by animal studies and smaller clinical studies, but to date, no large randomized clinical trials on the use of microcurrent therapy have been published. More recent clinical reports have also shown beneficial effects of ES on slowing the progression of RP but also lacks data from robust prospective clinical outcome studies. To our knowledge, this is the first prospective randomized study to evaluate the safety and clinical effectiveness of transpalpebral electrical stimulation (TpES) on the progression of RP.

**Methods:**

Randomized prospective study using N-of-1 trial 3 single-blind, crossover comparisons. The intervention period of each comparison is divided into treatment period and control period which are randomized arranged. Twelve participants will be strictly recruited in N-of-1 trial by the researcher in accordance with the inclusion and exclusion criteria. The main outcome of interest examined after each cycle of the 8-week intervention period is the assessment of the visual field (VF). Other variables of interest are best corrected visual acuity (BCVA), retinal function using electroretinogram (ERG), and visual function using NEI VFQ-25 questionnaire. Objective assessments of retinal changes will be undertaken using optical coherence tomography (OCT) and fundus autofluorescence (FAF).

**Discussion:**

The trial will evaluate the efficacy and safety of microcurrent stimulation on RP and provide high-quality evidence for clinical application through N-of-1 trial.

**Trial registration:**

Chinese Clinical Trial Registry; ChiCTR2300067357; https://www.chictr.org.cn/showproj.html?proj=174635. Registered on 5 January 2023

**Supplementary Information:**

The online version contains supplementary material available at 10.1186/s13063-024-07933-0.

## Introduction

Retinitis pigmentosa (RP) refers to a group of inherited retinal diseases that most often leads to severe visual impairment and blindness. Forms of RP and related diseases include Usher syndrome, Leber congenital amaurosis, and Bardet-Biedl syndrome among others. It is estimated that about 1.5 million people are affected by RP worldwide [1]. RP is the most common type of inheritable degenerative retinal disorder with an incidence of approximately 1/5000 ~ 1/3500 [2]. In China, the prevalence is about 1/4000, with the ratio of male to female is 3:2. RP is commonly divided into autosomal dominant, autosomal recessive, and X-linked inherited disease. It is characterized by progressive loss of retinal photoreceptor cells and retinal pigment epithelial (RPE) cells. The loss of rods photoreceptors precedes the loss of cone photoreceptors. The loss of photoreceptors results in the progressive impairment of night vision and of visual field (VF). Fundus examination shows typical “osteocytic” pigmentation, constriction of retinal vessels, and waxy pallor of optic disc [3]. Visual impairment develops typically already in the second or third decade of life with rapid progression to severe visual impairment before the age of 50, which leads to a serious negative impact on patient’s work and life, as well as economic and mental burden on family and society. The exact pathogenesis is unknown. A wide variety of experimental research is currently being conducted, such as possibilities for correcting the genetic defects of RP through gene carrier transfer [4], promoting the differentiation and maturation of RPE cells by stem cell transplantation [5], delaying the apoptosis of photoreceptor cells by neurotrophic factor [6], and inducing the electrical activity of retina and visual cortex by retinal prosthesis implantation [7]. These applications have yet to be applied clinically.

In 2004, when Chow and his team were investigating the safety and efficacy of the artificial silicon retina microchips implanted in the subretinal space, they discovered that low electric current could reduce and even reverse the loss of retinal function in RP, restoring the VF [5]. Since then, the application of electrical stimulation (ES) in the treatment of ophthalmic diseases has been widely studied. ES has shown to play a neuroprotective role in retinal diseases by improving both cerebral and ocular microcirculation. ES has shown to increase blood flow in both the optic nerve and retina [8, 9]. Studies have also shown that ES improves blood circulation of retinal choroid [10, 11], resulting in slowing the process of disease progression that ultimately leads to apoptosis of photoreceptor cells. ES has also shown to promote the growth of synapses and the survival of neurons, to activate retinal neurons to produce neural electrical activity, and to promote the transmission of photoelectric signals between neurons, resulting in the improvement of visual function [12, 13]. ES has been shown to stimulate glial cells to release neuroprotective factors, such as brain-derived neurotrophic factor and ciliary neurotrophic factor. Moreover, it can inhibit the expressions of tumor necrosis factor alpha and interleukin 1β, thus inducing apoptosis and inflammation. Therefore, there may exist multiple mechanisms for neuroprotection [14, 15].

In our own studies, we observed the effect of transpalpebral electrical stimulation (TpES) on both RP mice models and on RP patients had great potential to improve visual function. If applied properly, TpES may be a convenient and noninvasive therapy in regenerative medicine. To further verify the efficacy and safety of TpES treatment, we have designed this prospective N-of-1 single-blind, randomized controlled trial.

## Methods

### Objectives

The aim of this study is to evaluate the efficacy and safety of TpES in the treatment of RP and to provide high-quality evidence for clinical application through N-of-1 trial.

### Study design

**Fig. 1:**
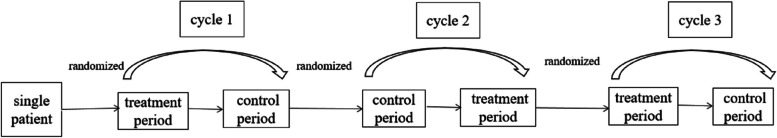
Example of N-of-1 trial design

This is a prospective, comparative, and single-blind N-of-1 trial, which includes 3 crossover comparisons. Each comparison includes an 8-week treatment period (A) and an 8-week control period (B). According to the random number generated by Strategic Applications Software (SAS), the random arrangement of AB or BA is presented in each cycle (Fig. 1).

#### Interventions

The operation procedure is as follows: (1) connect the device before preparing for treatment, and check whether the device is adequately powered. (2) Participants are asked to close their eyes. The electrodes and wet cotton pads soaked in pure water are then applied to the eyelids and the headband is fixed.

#### Study period of treatment (TpES intervention)

**Fig. 2:**
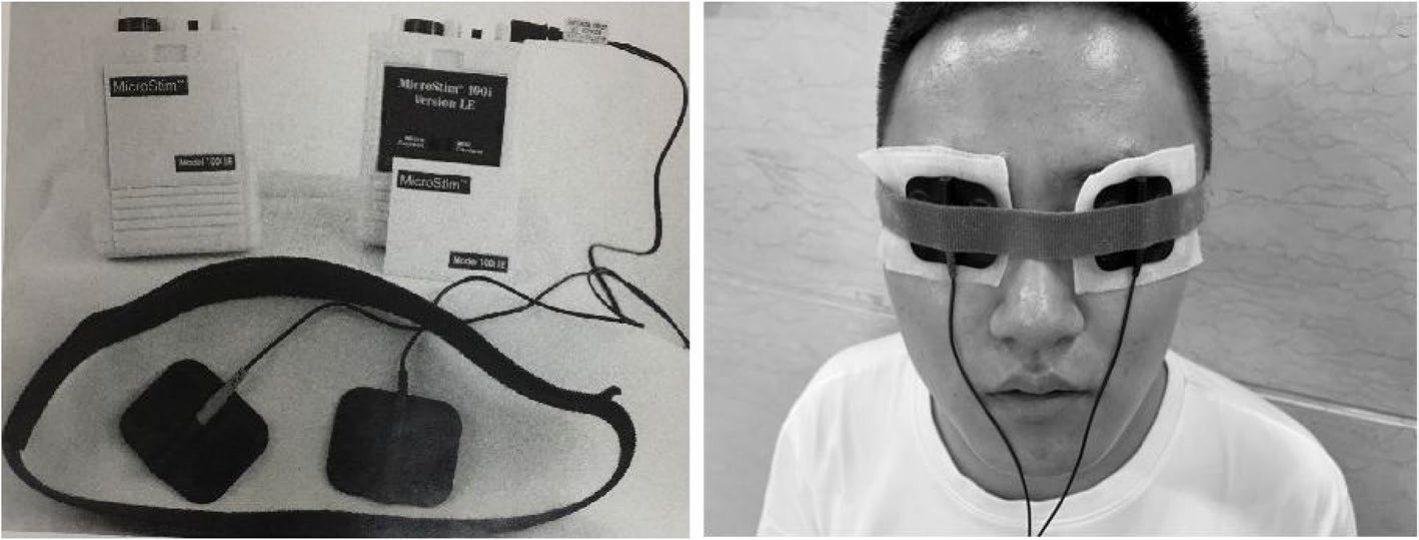
The microcurrent device and transpalpebral electrical stimulation

For the period of treatment, TpES is applied to both eyelids twice a day for 5 min each time. The current intensity is set to 0 ~ 800 μA. The 5-min protocol is comprised of a biphasic pulse with a frequency of 292 Hz for 30 s, 30 Hz for 30 s, 9.1 Hz for 2 min, and 0.3 Hz for 2 min. The participants should turn on the microcurrent device themselves and adjust the device’s current intensity knob so that the flash sensation just appears (Fig. 2).

#### Study period of control (no TpES intervention)

A TpES device is applied to the eyelids, but no electrical stimulation is given, and no pulsation is felt. Everything else remains the same.

During treatment and control periods, other treatments for RP will be discontinued, such as complementary therapies (herbal remedies and acupuncture) or other medications, throughout the study. Participants can take Western medicine to treat systemic diseases, such as hypertension or hyperlipidemia. Medication use will be recorded in detail in the case report form (CRF).

### Recruitment of research subjects

The subjects to be included for this study are RP patients recruited from the outpatient department of Eye Hospital, China Academy of Chinese Medical Sciences. This is a large hospital in Beijing, China, that sees many outpatients on a daily basis, offering both conventional treatment of eye diseases as well as traditional Chinese medicine and diverse energy treatments. RP is a rare eye disease. To enroll a sufficient number of participants, patients meeting the requirements for inclusion criteria were considered before the beginning of the study. The eligible patients will be notified as soon as the relevant procedures of the clinical trial are completed. Besides, we will post the recruitment posters through social media and other channels. Upon inclusion into the study, informed consent will be given with introduction of the details of the trial protocol including detail information about the treatment with its benefits and risks. Participants must sign an informed consent form prior to the study. Both the investigator and participants retained an informed consent form.

#### Eligibility

Inclusion criteria

1. Fulfill diagnostic criteria of RP
2. Aged between 18 and 80 years old
3. The best corrected visual acuity (BCVA) of at least one eye is greater than or equal to 35 letters detected on the ETDRS acuity chart (equal to 20/200 on Snellen chart)
4. VF radius of one eye is greater than 5° with the grayscale images of VF
5. Subjects agree to participate in the study and sign the informed consent form

Exclusion criteria

1. Patients with known adverse reactions to ocular ES
2. Patients with other eye diseases such as optic nerve diseases, glaucoma, macular edema not caused by RP, diabetic retinopathy, ocular autoimmune diseases, and ocular surface diseases affecting visual function
3. Patients who are considered by the investigators to be unable to provide reliable VF results in static or dynamic VF examinations. The value of reliability factor is greater than 15%
4. Mental illness or cognitive impairment and other disorders affecting research and follow-up
5. Patients with pacemakers
6. Participating in other clinical trials within the last 3 months and other therapies that affect the evaluation of efficacy
7. Pregnant and lactation women, and those who are hoping to become pregnant during the trial period

#### Termination criteria

1. If serious safety problems such as hospitalization occur in the trial and are judged to be treatment-related, the trial steering committee will stop the trial promptly
2. If there is serious deviation in the implementation of the clinical trial protocol, the data monitoring committee should discontinue the trial in time

#### Randomization and blinding

Allocation of cases to study groups is undertaken by a person not participating in the trial with the help of the generation of random sequencing using the SAS statistical software. Allocations are placed in sealed envelopes. Participants will be allocated an individual trial identification number. Research assistants will perform the allocation of patients to the study groups by opening of sealed envelopes at the time and prior to the first intervention cycle.

Due to the nature of the intervention, only the principal investigator is blinded to the treatment in this study. Unblinding is permissible only if the intervention is considered necessary to guide clinical management and with the consent of the principal investigator.

### Sample size

The sample size was calculated from SUN’s previous study [16]. The primary outcome was the change of mean defect (MD) value of VF after 8 weeks of treatment compared with baseline. Based on the reference (Sun et al.), the MD value of the treatment group is 2.06 ± 1.73. The MD value of the control group is 0.73 ± 1.93. The difference between the effect values of the treatment period and the control period was 1.43, and the standard deviation of the two periods was 1.93. When a two-tailed test with a test power of 80% and significance level of 5% was applied to the research, 10 cases were calculated [17]. Taking into account of 20% dropout rate, we ultimately identified 12 participants.

### Outcome measurements

When the participants are enrolled, their demographic data, such as name, gender, age, ethnicity, and contact details, are recorded. Medical histories are also recorded including current and prior diagnosis, course of RP disease, and any previous treatment.

#### Primary outcome

In this study, the primary outcome is VF [18]. Quantitative index of VF, including MD, mean sensitivity (MS), and square root of loss variance (sLV), will be examined by OCTOPUS automatic perimetry. The 32-test procedure is suitable for visual acuity greater than 0.3, and the LVC test procedure is suitable for visual acuity less than 0.3. The examination time points are before treatment and then at week 8, week 16, week 24, week 32, week 40, and week 48 (Table 1).

**Table 1:** Schedule of enrolment, intervention, assessment

Item	Screening	Observation period						
		Cycle 1		Cycle 2		Cycle 3		Closeout
Time point	Enrollment	Weeks 1–8	Weeks 9–16	Weeks 17–24	Weeks 25–32	Weeks 33–40	Weeks 41–48	
Demographic data	√							
Medical history	√							
Informed consent form	√							
BCVA	√	√	√	√	√	√	√	
VF	√	√	√	√	√	√	√	
OCT	√	√	√	√	√	√	√	
FAF	√	√	√	√	√	√	√	
ERG	√	√	√	√	√	√	√	
NEI VFQ-25	√	√	√	√	√	√	√	
Adverse event		√	√	√	√	√	√	

√ Items that must be performed

*BCVA* Best corrected visual acuity, *VF* Visual field, *OCT* Optical coherence tomography, *FAF* Fundus autofluorescence, *ERG* Electroretinogram, *NEI VFQ-25* 25-Item National Eye Institute Visual Function questionnaire

#### Secondary outcomes

Secondary outcomes include BCVA, optical coherence tomography (OCT), fundus autofluorescence (FAF), electroretinogram (ERG) and vision-related quality of life (VRQoL). The examination time points are before treatment and then at week 8, week 16, week 24, week 32, week 40, and week 48. BCVA will be examined by ETDRS visual acuity chart [19]. ERG includes five indexes, rod response, maximal combined rod-cone response, scotopic oscillatory potentials, cone response, and photopic 30 Hz flicker [20]. OCT measures the thickness of macular fovea [21]. Heidelberg ultra-wide angle photography technique will be used to examine the fundus images under the natural pupil [19]. The VRQoL is evaluated with NEI VFQ-25 questionnaire [22]. The NEI VFQ-25 consists of 26 items that address 12 aspects of daily living as follows: general health, general vision, near vision, distance vision, driving, peripheral vision, color vision, ocular pain, role limitation, dependency, social function, and mental health. Under the guidance of a research assistant, the questionnaire is self-administered by the study participants themselves.

### Safety evaluation

A standard examination will be performed to evaluate treatment safety at the beginning and the end of each period. The most common minor adverse events (AEs) from the ES procedures are skin allergies, headache, and dizziness. Other AEs such as unusual or uncomfortable sensations will be reported at the end of daily stimulation sessions.

### Statistical analysis

Statistical analysis will be performed by Statistical Package for the Social Sciences (SPSS) for version 25.0. Descriptive statistics will be used to analyze demographic characteristics of participants, including age and gender. Continuous variables will be presented as mean ± standard deviation. The primary and secondary outcomes will be analyzed using the full analysis set. Each patient’s paired trial is treated as an independent trial. For the in-group effects, the paired *t*-test is used for the data with normal distribution, and the Wilcoxon rank sum test is used for the data with non-normal distribution. For the intergroup effect, three pairs of data with normal distribution will be used with repeatability measure ANOVA test, and linear mixed-effects model will be used with non-normal distribution. For missing values, we will use a mixed-effects model for repeated measures. The *P* (2-tailed) value with threshold less than 0.05 will be considered statistically significant.

### Quality supervision

#### Management of study participants

We have prepared a reasonable recruitment plan for the convenience of research assistants and participants. For the communication with the study participants, we record participants’ WeChat account and mobile phone number. Especially in the control period, we communicate with participants regularly, know about their mental state and illness, and obtain maximum cooperation from participants. In addition, if any harm is associated with this clinical trial, compensation will be provided for the damage associated with the trial.

#### Training of research assistants

This study will unify the training to guide research assistants in their research work. Research assistants will master the standard operation of the microcurrent stimulation device, strictly follow the standard operating procedure, and minimize the error caused by the implementation process. For the Medical Examination Center and other related departments involved in the study, we have established a standard operating procedure, and a specific research assistant will be in charge of an item.

#### Data collection and management

The privacy of participants will be highly respected during the study. Participant’s details will be completely anonymized and stored on a secure database only for this trial and will not be disclosed without the permission of the participants. The results of all participants examined during the trial will be recorded in the CRF and be kept strictly confidential at Eye Hospital, China Academy of Chinese Medical Sciences. In addition, a database related to the study will be established, and an electronic case report form (e-CRF) will be used for data management. The information collected from study participants will remain confidential and will be anonymized for analysis purposes. To ensure the accuracy and confidential of the data information, all trial data checked by JXL and YJ are input into the e-CRF system, which will be manually reviewed. Research databases are secured with password-protected access systems. Only the principal investigator will have access to the final trial dataset. Any data required to support the protocol can be supplied on request. The data monitoring committee includes three members, KX, YML, and XYL, who are all professional research experts independent from the sponsor and competing interests. They will conduct interim analysis of the monitored data every 6 months and check the processing of the trial for quality supervision, but this does not constitute study termination. The ethics committee conducts follow-up review every yearly annual. The trial data will be published to enable international prospective meta-analyses from the corresponding author on reasonable request.

#### Roles and responsibilities

Lina Liang is both the principal investigator and sponsor; her contact information is lianglina163@163.com. The sponsor contributed to the design of the trial, who ensured that proper arrangements are in place to initiate, manage, and report on a study, and the decision to submit the report for publication.

#### Composition of the coordinating center and trial steering committee

The coordinating center includes WZ, JXL, and YJ who are responsible for the patient recruitment, follow-up, and data entry. WZ takes charge of the implementation of the research intervention, mainly recruiting and arranging follow-up participants. JXL and YJ are responsible for the data entry. The trial steering committee includes LNL and ZYH, who are mainly responsible for designing and supervising the trial protocol. The research administrators and the trial steering committee will meet over the course of the trial twice yearly to discuss the progress of the trial and review issues related to its implementation.

## Discussion

RP is a hereditary neurodegeneration of the retina. Multidisciplinary integration plays a positive role in promoting the treatment of this disease. A number of trials have been performed to develop an effective therapy to control the progression and complications of RP, including restoring defective genes and stem cell transplantation.

ES has been confirmed as a promising physical therapy which may recover visual functions in patients suffering from various ophthalmopathy, such as dry eye, glaucoma, traumatic optic neuropathy, retinal vein occlusion, RP, macular degeneration, and other eye diseases. The study demonstrated that transcorneal electrical stimulation (TcES) improved visual function of RP model in animal experiments, which was closely associated with the survival of the retinal ganglion cells and photoreceptors. Even more, the study showed that TcES was operated in clinical trials. However, TcES may lead to AEs, such as dry eye and foreign body sensation, whereupon Miura applied transdermal electrical stimulation therapy around the eyes and forehead and demonstrated the efficacy and safety without any adverse reactions of the cornea, skin, or facial nerve [23]. In order to balance the pros and cons, we bring forth new ideas for the therapy and decide to use TpES. Electricity deliveries to the eyes are via electrodes attached to the eyelids of subjects, without contacting with the cornea and conjunctiva. Compared with TcES, TpES has no side effects such as dry eye, punctate keratitis, and the risk of infection caused by the operation. Additionally, the device shows a non-invasive approach that can be performed by patients themselves. Based on clinical experience, TpES have proven to be effective and safe in Norway and other countries [24]. In our study, the four modulation frequencies are pre-set in the device. In general, it has been shown that a stimulation frequency of 20 Hz is ideal for stimulation. However, the optimal frequency for the use of retinal electrical stimulation has not been clearly established. Further research conducting varying stimulation frequency is needed with the potential to improve treatment methods for the treatment of ES.

Generally speaking, N-of-1 trial is a double-blind, randomized, multi-crossover trial with a single case as a control to evaluate the efficacy of the two therapies. N-of-1 trial belongs to RCT. In clinical practice, clinicians and patients pay more attention to the changes of curative effect after individuals taking medicine. This leads to RCT-based population evidence that may not be appropriate for individualized patients. Therefore, the research method for individual patient’s curative effect evaluation is indispensable. That is why N-of-1 came into being.

The clinical research is the first application in RP patients with the TpES therapy. With respect to the rationale for carrying out N-of-1 trial rather than RCT, we take the following reasons into consideration.

1. N-of-1 trials are randomized, prospective, controlled, multiple crossover trials in a single patient. Such trials are suitable for the following clinical applications: (1) chronic disease; (2) experimental treatments for rare diseases or with little or no previous research; (3) doubtful about the efficacy and safety of interventions for certain diseases; (4) the intervention has the characteristics of quick onset and short half-life period and the effect can disappear quickly after the intervention is stopped.
2. RP comprises a group of inherited retinal degenerative diseases with a prevalence of 1 in 4000, affecting 2 million persons globally. RP is associated with 3000 mutations in 100 genes. As a rare and chronic disease, adequate recruitment for group trials is not always feasible, informed consent is difficult to obtain, and retention is difficult too. Compared with RCT, the N-of-1 trial has higher power and requires smaller sample sizes.
3. RP is a highly heterogeneous disease. Studies have shown that genotype plays an important role in RP progression. Besides it, there are many other risk factors that may have effect on the progression of RP, such as inheritance patterns, smoking, physical activity, and other demographic and environmental factors. Due to the small cohort sample size and the large clinical heterogeneity of RP patients, it is difficult to obtain high-quality evidence of therapeutic effectiveness. With the consideration of heterogeneity, N-of-1 trial is a more suitable choice.
4. There have been no reports of transpalpebral electrical stimulation for RP treatment up to now. It is an individualized treatment, and the intensity of stimulating current varies with the condition of the retinopathy in each patient. In the trial, patients will perform the treatment by themselves. Therefore, N-of-1 trial has better feasibility than conventional RCT. Moreover, the intervention has the characteristics of quick onset, and the effect can disappear relatively quickly after the intervention is stopped, which make it suitable for N-of-1 trial.

Taken together, based on the characteristics of RP and the intervention method, it is very suitable for the study of RP.

This trial has two strengths. First, RP is a rare clinical eye disease with a low prevalence. The patients are required to undergo three cycles of crossover trial, so the sample size included in this study is small. N-of-1 trial is precisely suitable for studying RP. Second, the research method of N-of-1 trial is relatively flexible. During each cycle, the effect of treatment can be directly evaluated by observing and comparing patients in two periods. There is no necessity to wait until the end of the trial to make a statistical analysis of relevant data.

A limitation of the trial is that it requires multiple periods of observation. Therefore, the trial usually last longer than common RCTs.

The lack of long-term benefits of patients is another potential limitation. It is known that RP is a chronic and progressive eye disease. Our study protocol does not carry out the follow-up period, and the follow-up effect of patients cannot be observed.

## Trial status

The updated version of trial protocol (version 2.0, 1 December 2022) has been reviewed and approved on 23 December 2022 (YKEC-KT-2022–043-P002). Recruitment is start in January 2023 and is expected to finish in March 2024.

### Supplementary Information

**Additional file 1.** SPIRIT checklist for Trials.

**Additional file 2.** Medical ethics review approval.

## Data Availability

The datasets analyzed during the current study are available from the corresponding author on reasonable request, as is the full protocol.

## Abbreviations

AEs: Adverse events
BCVA: Best corrected visual acuity
CRF: Case report form
e-CRF: Electronic case report form
ERG: Electroretinogram
ES: Electrical stimulation
FAF: Fundus autofluorescence
MS: Mean sensitivity
MD: Mean defect
OCT: Optical coherence tomography
RP: Retinitis pigmentosa
RPE: Retinal pigment epithelial
SAS: Strategic Applications Software
sLV: Square root of loss variance
SPSS: Statistical Package for the Social Sciences
TcES: Transcorneal electrical stimulation
TpES: Transpalpebral electrical stimulation
VF: Visual field
VRQoL: Vision-related quality of life
