# Supplementary material for: Transpalpebral electrical stimulation for the treatment of retinitis pigmentosa: study protocol for a series of N-of-1 single-blind, randomized controlled trial
Source: Trials. 2024 Jan 27;25:89. doi: 10.1186/s13063-024-07933-0 (PMC10821291; doi:10.1186/s13063-024-07933-0)
Supplement: Supplementary file 2 — Additional file 2. Medical ethics review approval. [file 13063_2024_7933_MOESM2_ESM.doc]

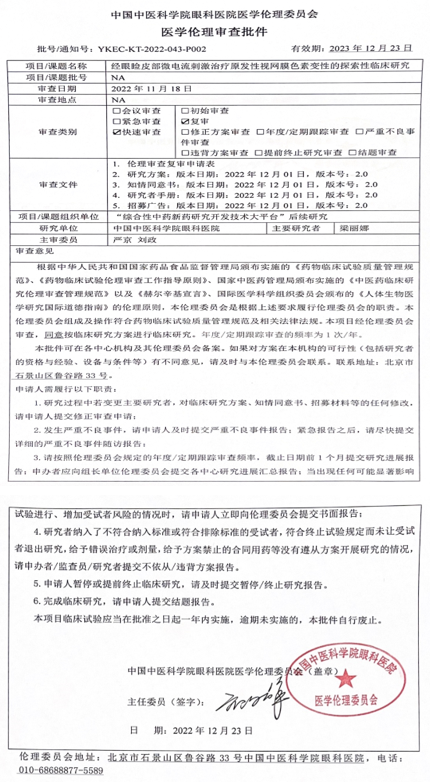


Medical Ethics Committee, Eye Hospital, China Academy of Chinese Medical Sciences

**Medical ethics review approval**

Batch Number/NotificationNumber :YKEC-KT-2022-043-P002 Expiration Date: December 23, 2023

| Project Name | An exploratory clinical study of primary retinitis pigmentosa by transpalpebral electrical stimulation | | | |
| --- | --- | --- | --- | --- |
| Project Batch Number | NA | | | |
| Review Date | November 18, 2022 | | | |
| Review Place | NA | | | |
| Review Category | ¨Conference Review  ¨Urgent Review  þExpedited Review | ¨Initial Review  þReview  ¨Revision Protocol Review ¨Annual/Regular Follow-up Review ¨Serious Adverse Events Review  ¨Non-compliance with Protocol Review ¨Early Termination of Study Review ¨Conclusion Review | | |
| Review Document | 1. 1. Application for ethics review   2. Research Protocol : Version date: December 01, 2022, version number: 2.0  3.Informed Consent Form: Version date: December 01, 2022, version number: 2.0  4. Investigator’s Brochure: Version Date: December 01, 2022, version number: 2.0  5. Recruitment Advertisement: Version date: December 01, 2022, version number: 2.0 | | | |
| Project/Topic Organization Unit | "Comprehensive Chinese Medicine New drug research and development technology platform" follow-up study | | | |
| Research Unit | China Academy of Chinese Medicine Eye Hospital | | Principal investigator | Lina Liang |
| Presiding Member | Jing Yan Zheng Liu | | | |
| Review Opinion | | | | |
| In accordance with "Good Practice for the Quality Management of Drug Clinical Trials" and "Guiding Principles for the Municipal Investigation of Drug Clinical Trial Ethics" promulgated by the State Drug and Food Administration of the People's Republic of China, "Good Practice for the Management of the Ethical Review of Traditional Chinese Medicine Clinical Research" promulgated by the State Administration of Traditional Chinese Medicine and the "Declaration of Helsinki", The ethical principles of the International Ethical Guidelines for Human Biomedical Research issued by the International Organization Committee for Medical Sciences are adopted. The Ethics Committee performs the duties of the Ethics Committee according to the above requirements. The composition and operation of the Ethics Committee comply with the quality management standards for drug clinical trials and relevant laws and regulations. This project has been reviewed by the Ethics Committee, and the response is to conduct clinical research according to the clinical research protocol, and the annual/regular follow-up review rate is once a year.  This approval document can be recorded in each central institution and its ethics committee. If you have different opinions on the feasibility of the program in this institution (including the qualifications and experience of the researcher, equipment and conditions, etc.), please contact the Ethics Committee in time, contact address: 33 Lugu Road, Shijingshan District, Beijing.  The applicant shall perform the following duties:  1. If the main investigator is changed during the research process, any modification to the clinical research protocol, informed inquiry letter, phase screen materials, etc., shall be submitted to the applicant for amendment of the market investigation application.  2. Serious adverse events occur. Please submit a serious adverse event report in a timely manner: After the emergency report, please submit a detailed serious adverse event follow-up report as soon as possible.  3. Please follow the annual/regular frequency of market inspection stipulated by the Ethics Committee and submit the research progress report one month before the deadline: the central office shall submit the summary report of the research progress of each center to the ethics committee of the group leader. When there is any possible event that the trial is conducted and the risk to the subject is increased, the applicant is requested to immediately submit a written report to the Ethics Committee  4. If the investigator enrolled subjects who did not meet the inclusion criteria or meet the exclusion criteria, did not withdraw the subjects from the study while meeting the trial termination requirements, gave the wrong treatment or dose, gave the contracted medication prohibited by the protocol, etc., the investigator/monitor/investigator was requested to submit a non-compliance/violation report.  5. If the applicant discontinues or prematurely terminates the clinical study, please submit the study suspension/termination report in time.  6. Complete the clinical study and ask the applicant to submit the final report.  The clinical trial of this project shall be implemented within one year from the date of approval. If it is not implemented within the time limit, this approval document shall be annulled automatically.  Medical Ethics Committee, Eye Hospital, China Academy of Chinese Medical Sciences(Seal)  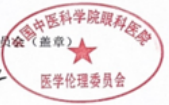  Signature of the Chairman: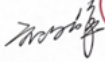    Date: December 23, 2022 | | | | |

Ethics Committee Address: Eye Hospital, China Academy of Chinese Medical Sciences, 33 Lugu Road, Shijingshan District, Beijing, China, Tel: 010-68688877-5589
